# Aortic Thrombosis and Acute Limb Ischemia Secondary to COVID Hypercoagulability

**DOI:** 10.7759/cureus.16171

**Published:** 2021-07-04

**Authors:** Nicholas B Burley, Paul S Dy, Shreyas Kalantri, Kanwal Razzaq

**Affiliations:** 1 Internal Medicine, Sinai Hospital of Baltimore, Baltimore, USA

**Keywords:** covid 19, arterial thrombus occlusion, covid-19, aorta thrombosis, aortic thrombosis, acute limb ischemia, covid hypercoagulability, covid-related hypercoagulability, thrombosis, coronavirus

## Abstract

Severe acute respiratory syndrome coronavirus 2 (SARS-CoV-2) or coronavirus disease 2019 (COVID-19), first identified in December 2019 in Wuhan, China, has rapidly spread worldwide, is now a public health emergency, and has been declared a pandemic. While SARS-CoV-2 is known to cause significant pulmonary disease, ranging from pneumonia to acute respiratory distress syndrome (ARDS), various extrapulmonary manifestations of COVID-19 have also been reported. Growing evidence suggests that COVID-19 leads to a hypercoagulable state leading to micro and macro-vascular angiopathies. We present a case of an 80-year-old male without a previous history of prothrombotic disorders who developed descending aortic thrombosis, approximately 40% stenosis, at the level of the diaphragmatic hiatus and acute limb ischemia secondary to COVID-19 requiring emergent surgical intervention. After 12 days of persistent ischemic left lower extremity imaging despite thrombectomy, bypass, and therapeutic heparin, the patient's limb was deemed non-salvageable and underwent left above-knee amputation. Transthoracic echocardiogram revealed normal left ventricular function, moderate pulmonary hypertension, and no evidence of atrial septal defect, aortic root abnormalities, or intraventricular thrombi. Evaluation of autoimmune and inflammatory vasculitis was negative. While further study into the prothrombotic nature of this condition still needs to be pursued, the thromboembolic risk of COVID-19 represents an urgent need for appropriate anticoagulation for venous thrombosis. Arterial thrombosis requires other kinds of management to avoid the severe adverse effects of emboli and related ischemia. This current case highlights the need for randomized control trials testing different prophylactic strategies. Further evidence is also required for the role of amputation surgery when initial interventions for revascularization fail to restore blood flow.

## Introduction

Severe acute respiratory syndrome coronavirus 2 (SARS-CoV-2) or coronavirus disease 2019 (COVID-19), first identified in December 2019 in Wuhan, China, has rapidly spread worldwide, is now a public health emergency, and has been declared a pandemic [1].

SARS-CoV-2 is known to cause significant pulmonary disease, ranging from pneumonia to acute respiratory distress syndrome (ARDS) although various extrapulmonary manifestations of COVID-19 have also been reported [2]. Increasing evidence suggests that COVID-19 leads to a hypercoagulable state, leading to micro and macro-vascular angiopathies. COVID-19 leads to an increase in D-Dimer, prothrombin, and fibrinogen level further supports the finding [3-4]. Moreover, a severe form of COVID-19 can lead to systemic hyperinflammation with an increase in various pro-inflammatory cytokines such as tumor necrosis factor, interleukin (IL)-6, and IL-1β could lead to a pro-thrombotic state [5-6]. There is significant evidence suggesting venous thrombotic events. However, currently few reports suggest intra-arterial thrombotic complications [7].

We present a case of an 80-year-old male who developed aortic thrombosis and acute limb ischemia secondary to COVID-19 requiring emergent surgical intervention.

## Case presentation

**Figure 1:**
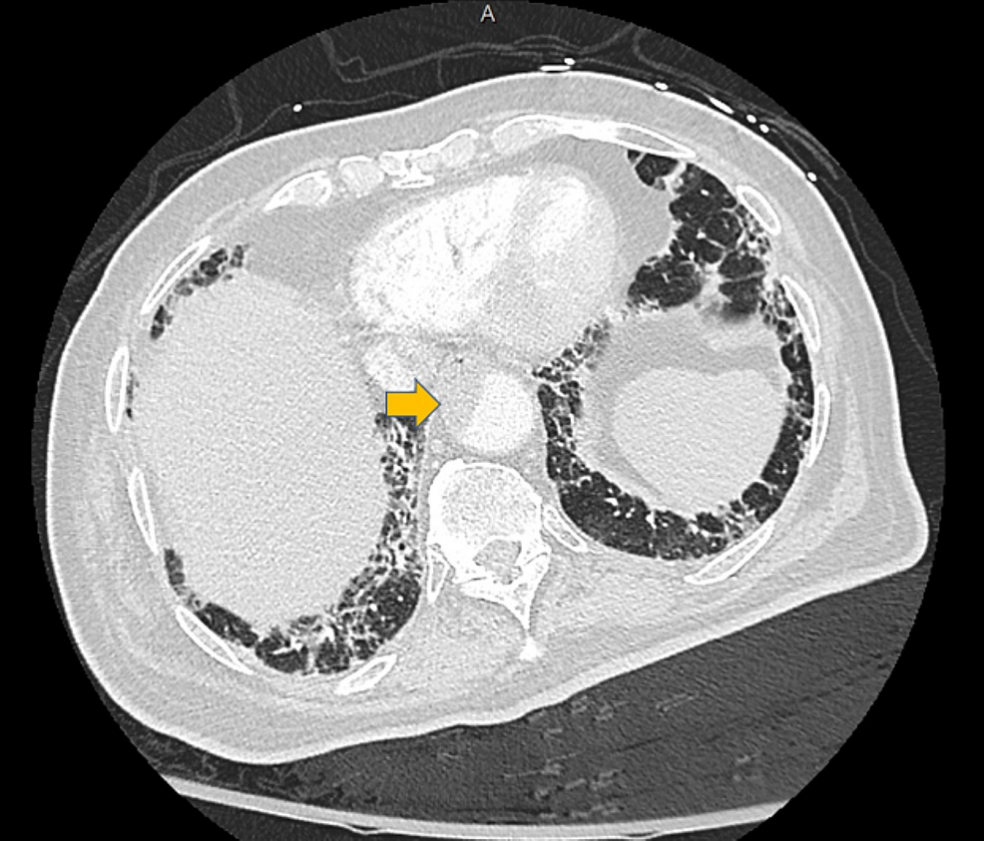
Computed tomography with intravenous contrast of chest Focal 6.2 cm intraluminal thrombus narrowing 40% of the aorta at the level of the diaphragmatic hiatus

An 80-year-old male with a past medical history of heart failure with preserved ejection fraction (HFpEF), hypertension, and hyperlipidemia presented with cough and dyspnea for two weeks and was found to have COVID-19 infection. On hospital Day 1, the patient developed acute left ankle pain and coolness, with fifth toe discoloration. Emergent arterial duplex ultrasound confirmed an occluded left lower extremity artery, including partial occlusion to the level of external iliac artery and complete occlusion at the common femoral artery level. Continuous infusion heparin anticoagulation was initiated and vascular surgery was consulted. A review of CT-thorax pulmonary embolus (PE) protocol revealed a focal 6.2 cm intraluminal thrombus narrowing the aorta approximately 40% at the level of the diaphragmatic hiatus (Figure 1).

The patient was emergently taken to the operating room (OR) for thrombectomy, femoral-popliteal bypass via groin cutdown, femoral endarterectomy, dorsalis pedis and posterior tibial exploration, and medial and lateral fasciotomy and subsequently admitted to the surgical ICU (SICU). The patient was managed in the SICU with continuous heparin infusion, packed red blood cell transfusion (nadir hemoglobin 6.3 mg/dL), azithromycin, ceftriaxone, methylprednisolone, convalescent plasma, and tocilizumab for severe COVID-19 infection. A transthoracic echocardiogram left ventricular (LV) ejection fraction of 60%-65%, impaired LV relaxation, moderate pulmonary hypertension (peak pulmonary systolic pressure of 55 mmHg), and normal right ventricular size and function. No atrial septal defect, intraventricular thrombi, or aortic root abnormalities were detected.

Due to persistent ischemic left lower extremity imaging despite thrombectomy and bypass, the patient’s limb was deemed non-salvageable and underwent left above-knee amputation on postoperative Day 12. The patient was safely transitioned from heparin to warfarin anticoagulation in preparation for discharge.

During hospitalization, further evaluation of other autoimmune and inflammatory vasculitis etiologies was negative, including c-antineutrophil cytoplasmic antibodies (c-ANCA), p-ANCA, ANCA proteinase 3 (PR3), ANCA myeloperoxidase (MPO), and ACNA atypical. The patient had no personal or family history of vasculitis or prothrombotic conditions. Further workup for prothrombotic coagulation disorders was not pursued during hospitalization. Of note, the patient’s son was also hospitalized for COVID-19 infection and was discharged several days later while anticoagulated for a self-described ‘blood clot,’ although it is unclear if it was arterial or venous thrombosis.

The outcome for this gentleman was a safe discharge to a skilled nursing facility with close follow-up with vascular surgery, physical medicine and rehabilitation, and internal medicine post-hospitalization. A follow-up review shows that the patient is recovering well and is continuing to undergo physical and occupational therapy, pain management, and immediate care by the surgical team in the outpatient setting.

## Discussion

The COVID-19 virus has been documented to have numerous manifestations and deleterious effects on the cardiovascular, renal, and pulmonary systems. This case continues to build upon the growing knowledge of the prothrombotic and inflammatory effects of the COVID-19 virus.

While previous cases have discussed the effects on the venous system and the increased likelihood of both deep venous thrombosis and pulmonary emboli, this case raises concern about the effect on the arterial system’s capability of producing thrombi and resulting ischemic tissue damage from emboli. As we learn more about the relationship between venous and arterial thrombosis, we also recognize the increased risk of clotting in both vascular systems from COVID-19 [8].

There have been several case reports identifying arterial thrombosis and related ischemia in healthy adults without risk factors as a result of a COVID-19 infection [9]. Additionally, the hypercoagulable state presents unique challenges, as preliminary data showed unexpected resistance to revascularization techniques [10].

Several potential risk factors for thrombosis have been proposed, including hypoxemia, endothelial dysfunction, and inflammation, though the exact pathologic mechanism is still yet to be known [11]. It is also unclear if the endothelial injury is a result of SIRS or caused directly by COVID-19 [12].

A study conducted by Tang et al. showed that abnormal coagulation results, especially the markedly elevated D-dimer level and fibrin degradation products, are common in patients who have died of COVID-19-related pneumonia [13]. Another study by Han et al. showed that blood coagulation function is deranged compared with healthy uninfected people. Monitoring coagulation parameters, such as D-dimer, fibrin degradation products, and fibrinogen, could enable early identification of severe cases and routine monitoring could also help predict disease progression [14].

Early anticoagulation could benefit patients with COVID 19. We are well aware that heparin is an effective prophylactic measure to prevent venous thromboembolism [15]. Apart from the fact that heparin has competitive binding activity against coronavirus, it has also been shown to have suppressive activity against the development of cytokine storm, which is a pathogenetic process of COVID-19 resulting in acute lung injury and death [16-18].

The historically associated risk factors for VTE appear to increase the risk of arterial thrombi while additional factors raise the likelihood of arterial thrombi significantly such as vasculitis and autoimmune conditions. A new risk for arterial thrombosis appears to be COVID-19 [8].

## Conclusions

In summary, COVID-19, with its propensity to induce hypercoagulability, can affect not just the venous system but the arterial as well; it has the potential to cause aortic thrombosis as demonstrated in this case. Prompt anticoagulation is necessitated in such conditions because of the risk for debilitating and life-threatening ischemia. Further studies are needed to evaluate preventative strategies and amputation surgery when initial interventions for revascularization fail to restore blood flow.
